# Digital Cranial Endocast of *Hyopsodus* (Mammalia, “Condylarthra”): A Case of Paleogene Terrestrial Echolocation?

**DOI:** 10.1371/journal.pone.0030000

**Published:** 2012-02-10

**Authors:** Maeva J. Orliac, Christine Argot, Emmanuel Gilissen

**Affiliations:** 1 Université Montpellier 2, ISE-M UMR 5554, Montpellier, France; 2 Muséum National d'Histoire Naturelle, UMR 7207 CR2P, Paris, France; 3 Royal Museum for Central Africa, Department of African Zoology, Tervuren, Belgium; 4 Laboratory of Histology and Neuropathology, Université Libre de Bruxelles, Brussels, Belgium; 5 Department of Anthropology, University of Arkansas, Fayetteville, Arkansas, United States of America; University College London, United Kingdom

## Abstract

We here describe the endocranial cast of the Eocene archaic ungulate *Hyopsodus lepidus* AMNH 143783 (Bridgerian, North America) reconstructed from X-ray computed microtomography data. This represents the first complete cranial endocast known for Hyopsodontinae. The *Hyopsodus* endocast is compared to other known “condylarthran” endocasts, i. e. those of *Pleuraspidotherium* (Pleuraspidotheriidae), *Arctocyon* (Arctocyonidae), *Meniscotherium* (Meniscotheriidae), *Phenacodus* (Phenacodontidae), as well as to basal perissodactyls (*Hyracotherium*) and artiodactyls (*Cebochoerus*, *Homacodon*). *Hyopsodus* presents one of the highest encephalization quotients of archaic ungulates and shows an “advanced version” of the basal ungulate brain pattern, with a mosaic of archaic characters such as large olfactory bulbs, weak ventral expansion of the neopallium, and absence of neopallium fissuration, as well as more specialized ones such as the relative reduction of the cerebellum compared to cerebrum or the enlargement of the inferior colliculus. As in other archaic ungulates, *Hyopsodus* midbrain exposure is important, but it exhibits a dorsally protruding largely developed inferior colliculus, a feature unique among “Condylarthra”. A potential correlation between the development of the inferior colliculus in *Hyopsodus* and the use of terrestrial echolocation as observed in extant tenrecs and shrews is discussed. The detailed analysis of the overall morphology of the postcranial skeleton of *Hyopsodus* indicates a nimble, fast moving animal that likely lived in burrows. This would be compatible with terrestrial echolocation used by the animal to investigate subterranean habitat and/or to minimize predation during nocturnal exploration of the environment.

## Introduction

A major function of the brain is the maintenance of physiological condition (homeostasy). By collecting and processing sensory information, it insures optimal behavioral responses in a given environment. Therefore, several brain structures are molded by the ecophysiological situation of the animal within its niche [1]–[4]. The increased use of computed tomography (CTscan) recently gave an unprecedented access to internal structures of fossils, and triggered new interest for vertebrate cranial endocast studies (e.g., [5]–[14]). These recent works are building upon fundamental studies performed during the second half of the 20^th^ century (e.g., [15]–[20]) and cranial endocast anatomy is now documented for major mammalians clades.

The brain and associated soft tissues such as meninges, nerves, veins and arteries are sheltered in the neurocranium and leave imprints on its tabula interna. The study of the endocranial cast is the only means to reach an understanding about the nervous and sensory system of extinct species. Edinger [21] and Jerison [17] demonstrated that analysis of mammalian endocasts can provide a good approximation of the relative size of the major encephalic subdivisions and accordingly, comparative studies of fossil mammalian endocasts largely contributed to our knowledge of brain evolutionary history (e.g., [6], [17], [22]–[25]).

**Figure 1:**
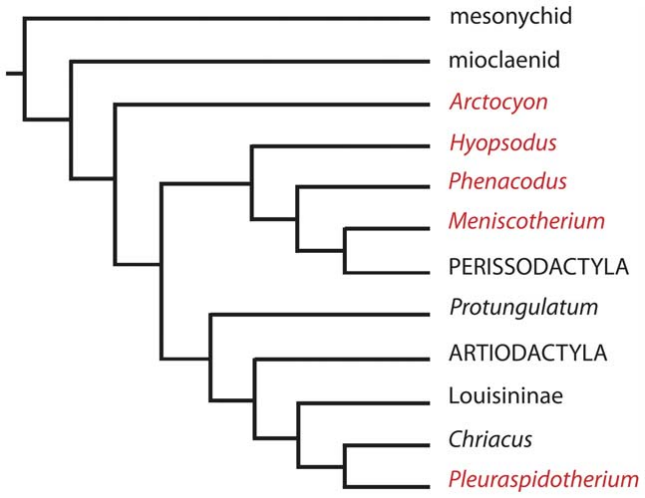
Simplified phylogenetic relationships among basal ungulates, modified after [**36, fig. 11B**].

In the history of mammals, ‘Condylarthra’ consists of a para – or polyphyletic assemblage of Paleocene-Eocene early diverging ungulates that potentially played a crucial role in the onset of crown ungulate clades such as Perissodactyla, Artiodactyla, Cetacea, Proboscidea, Sirenia, and Hyracoidea (e.g., [26]–[29]). The braincase of several groups of these early diverging ungulates has been described in the literature and major families are documented: Arctocyonidae (*Arctocyon*, [30], [31]; *Artocyonides*, [31]), Phenacodontidae (*Phenacodus*, [15], [30], [32], [33]; *Meniscotherium*, [34]), Periptychidae (*Periptychus*, [30]), Hyopsodontidae (*Hyopsodus*, [35]), and Pleuraspidotheriidae (*Pleuraspidotherium*, [31]). The diversity of these groups is also reflected in the disparity of brain morphologies. The phylogenetic relationships of archaic ungulates remain a current topic of investigation. A recent hypothesis of relationships among basal ungulates mammals [36] including most of the ‘condylarthran’ taxa for which the endocranial cast is known is presented in figure 1.

In this work, we describe the endocast of *Hyopsodus* following 3D virtual reconstruction based on CT scan data. *Hyopsodus* is the most common mammal in several Early and Middle Eocene faunas from western North America ([35], [37]). It first appeared in the North American record in the early Wasatchian (or latest Clarkforkian, i.e. around the Paleocene-Eocene transition) and is recorded until the late early Chadronian (latest Eocene, [37], [38]). Gazin [35] provided the most extensive morphological revision of the genus. He described the endocranial features of *Hyopsodus* based on a partial cranial endocast of *H. miticulus* (USNM 23745) reconstructed from assembled cranial fragments of the same individual originating from Wyoming (Knight Member, Wasatch Formation, Green River Basin). This reconstruction documents the cerebellum and the midbrain as well as the posterior part of the telencephalon. A natural cast of the olfactory lobes of the same species is also described by the same author [35]. The ventral portion of the endocranial cavity of *Hyopsodus* is known from a specimen of *H. paulus* from the Bridger Formation (late Early, early Middle Eocene, Wyoming) in which the base of the endocranium was exposed [35]. The endocranial structures of *Hyopsodus* are thus only partially known from Gazin's work, and from fragmentary individuals belonging to distinct species. The relative proportions of the various brain parts, especially most of the forebrain including the extension of the neopallium, remained undescribed. In this study, we document the first complete endocranial cast of *Hyopsodus*, bringing crucial information about the relative proportions of the various encephalic structures. We detail forebrain morphology and sinuses network, and compare the endocast of *Hyopsodus* to those of other early diverging ungulates. The peculiar structure of endocranial cast of *Hyopsodus* is compared to its postcranial anatomy and used to discuss its ecology, especially its putative ability of using terrestrial echolocation to progress in burrows or to avoid nocturnal predators.

## Results and Discussion

### Digital cranial endocast of *Hyopsodus*

**Figure 2:**
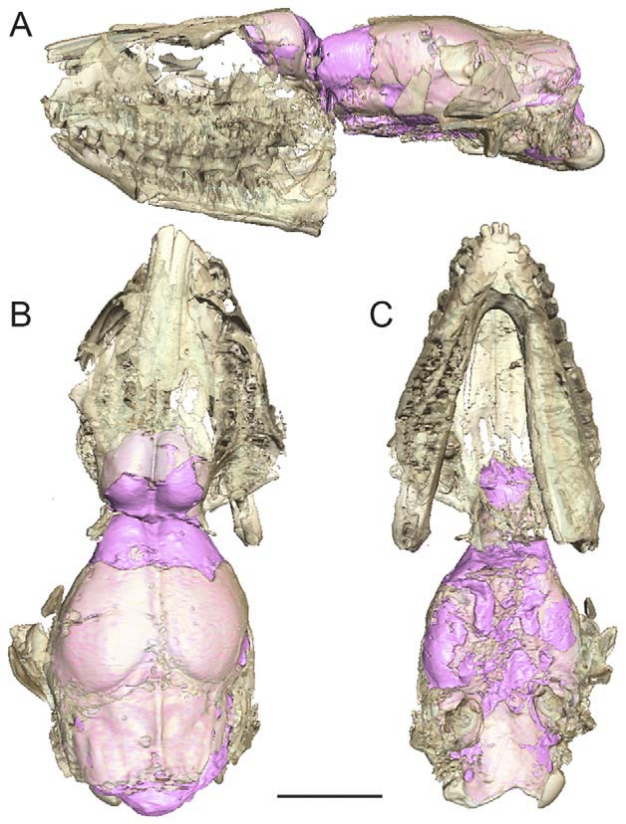
In situ endocast of *Hyopsodus lepidus* (AMNH 143783). Specimen illustrated in lateral (A), dorsal (B), and ventral (C) views. Scale bar = 1 cm.

**Figure 3:**
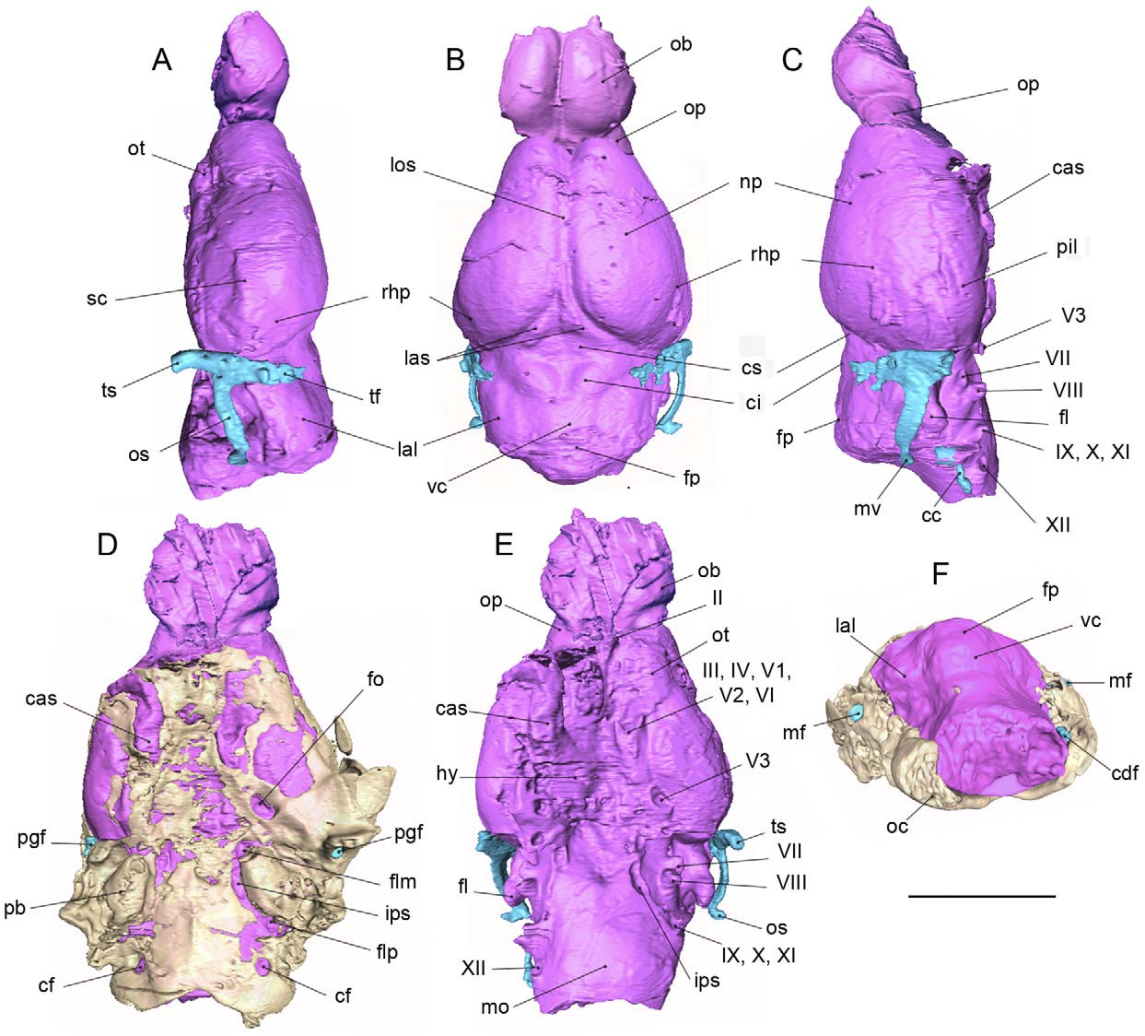
Labelled endocast and basicranium of *Hyopsodus lepidus* (AMNH 143783). Reconstruction illustrated in left lateral (A), dorsal (B), right lateral (C), ventral with basicranium (D), ventral without basicranium (E), and posterior views. Abbreviations: cas, cavernous sinus; cc, condyloid canal; cdf, condyloid foramen; ci, colliculi inferior; cf, condylar foramen; cs, colliculi superior; fl, paraflocculus; flm, foramen lacerum medium; flp, foramen lacerum posterius; fo, foramen ovale; fp, fissure prima; hy, hypophysis; ips,inferior petrosal sinus; lal, lateral lobe of cerebellum; las, lateral sinus (or transverse sinus); los, longitudinal sinus; mf, mastoid foramen; mo, medulla oblongata; ms, mesencephalon; mv, mastoid vein; np, neopallium; ob, olfactory bulb; oc, occipital condyle; op, olfactory peduncle; os, occipital sinus; ot, olfactory tubercle; pb, petrosal bone; pgf, postglenoid foramen; pil, piriform lobe; rhp, rhinal fissure; sc, sinusal canal; tf, temporal foramen; ts, temporal sinus; vc, vermis cerebelli. Scale bar = 1 cm.

The braincase of AMNH 143783 is broken in two parts at the base of the olfactory bulbs. The two fragments match perfectly and the neurocranium is complete (Figure 2). The skull has undergone a slight dorso-ventral compression and the braincase is slightly deformed by a lateral shift. Measurements of the endocast are provided in Table 1. The various parts of the brain, olfactory bulbs, cerebral hemisphere and cerebellum are aligned within the same plane and serially arranged (Figure 3A, C). The vermis of the cerebellum constitutes the highest point of the brain. The cerebrum is relatively flat compared with the cerebellum. The shape of the cerebrum is correlated to the almost complete absence of telencephalic flexure. The maximal width is at the level of the piriform lobes (Figure 3B). The development of the olfactory peduncles and bulbs is important and their length represents approximately half of the total length of the cerebrum (Figs. 2B, 3B). The forebrain length represents approximately 2/3 of the total length of the endocast (Figure 3B).

**Table 1:** Endocast measurements for *Hyopsodus* (given in mm, and mm^3^ for endocast volume).

	AMNH 143783	*Hyopsodus* (Gazin, [35])
Endocast volume	2790	-
Endocast flexure (estimated)	30°–35°	-
Endocast maximum length	32.37	-
Olfactory bulb cast anteroposterior length	R:7.47; L:7.17	8.5*
Olfactory bulb cast height	R:5.66; L:5.05	-
Olfactory bulb cast maximal width	9.01	9.5*
Cerebrum cast anteroposterior length	13.86	-
Cerebrum cast maximal width	18.11	-
Cerebrum height	R:10.89; L: 9.04	15.05**
Cerebellum cast anteroposterior length	6.77	8.00**
Cerebellum cast height	12.11	15.00**

R = right side of specimen; L = left side of specimen;

* = *H. paulus* ([35], pl. 1);

** = *H. miticulus* ([35], pl. 5).

#### Rhinencephalon

The specimen AMNH 143783 gives access to the complete extension of the rhinencephalon. The latter is strongly developed, as it almost represents half of the forebrain. The bulging olfactory lobes are of large size. Their combined diameters equal the width of the dorsal roof of the skull in the posterior half of the orbits (Figure 2B). The two lobes are elongated and contiguous. They are prolonged by long olfactory fibers spreading into the anterior part of the orbits and beyond (not reconstructed on the 3D model because of bad preservation of the specimen). The olfactory bulbs directly extend the olfactory peduncles. Both structures are aligned with the rest of the telencephalon. The location of the olfactory tubercles is not clear on the right side of the skull because of postmortem deformation. It is observable on the left side, although weakly prominent (Figure 3A). The piriform lobes represent most of the cerebral hemispheres; they are visible in dorsal view in the posterolateral part of the cerebrum where they constitute the greatest width of the brain. They are dorsally delimited by the posterior rhinal fissure located in the dorsal third of the cerebrum height. A sinus might be present in the posterolateral part of the piriform lobe, but there is no trace of its anterior extension.

#### Neopallium

In the description of the *Hyopsodus* braincase provided by Gazin [35], a considerable portion of the forebrain was missing and the telencephalon was only partially described. Especially, the location of the rhinal fissure was unknown. In the specimen described here, the posterior rhinal fissure is shallow but clearly present on both sides of the telencephalon (Figure 3 A–C). It is located in the dorsal third of the hemisphere and delimits the posteroventral border of the neopallium. The anterior rhinal fissure is difficult to locate. When present, it is limited to the anteriormost part of the cerebrum and does not abut on the posterior rhinal fissure. The neopallium thus forms a reduced cap on the cerebral hemisphere. The neopallium is smooth or lissencephalic, suggesting that there were no gyri or sulci (neither the lateral sulcus nor the suprasylvian sulcus). Only a subtle convexity lays on the dorsoposterior part of the neopallium, slightly posterior to the location of the gyrus 3 described in earliest artiodactyls ([16]; Orliac pers. obs). This structure marks the highest point of the cerebrum, with its dorsal part being flat and anteriorly sloping. The neopallium is distinctly narrower in its anterior portion. Between the medial margins of the neopallium, the longitudinal sinus in *Hyopsodus* is deep and clearly sets apart the two cerebral hemispheres. The lateral or transverse sinuses separate just ahead of the superior colliculi.

#### Midbrain

The posterior limit of the neopallium is far apart from the lobes of the cerebellum, and the tectum of the midbrain is broadly exposed on the dorsal surface. The midbrain exposure is accentuated by the anterior divergence of the two cerebral hemispheres.

The corpora quadrigemina (colliculi) are clearly visible. The inferior colliculus is especially important, and its development occupies most of the exposed midbrain. This special feature is also observed in the specimen described by Gazin [35]. On x-ray radiographies, quasi perpendicular to the tabula interna, the tentorium cerebelli is a thick crest of bone separating the cerebrum from the cerebellum.

#### Cerebellum

The cerebellum is large and exhibits a well delimited vermis, cerebellar hemispheres (lateral lobes) and paraflocculus. The vermis of the cerebellum is wide and elevated; its dorsal extension is pointed and reaches the same level as the dorsal face of the telencephalon. The fissura prima is located in the posterior third of the vermis. The cerebellar hemispheres are separated from the cerebrum by a broad and shallow paramedian fissure. The former are moderately developed and not inflated, sloping anteriorly. Their dorsal surface is almost flat. In dorsal view, their posterior extension does not reach that of the vermis, with the posterior part of the latter forming a wide bulge. On both ventrolateral surfaces, the paraflocculi are observable. They consist of two rounded and large sized lobes that project outwards, downwards and somewhat backwards.

#### Nerves and sinuses

The superior petrosal sinus is located underneath the cerebellar hemispheres, a continuation of the transverse or lateral sinus system. The posterior part of the sinus system cannot be described because most of the occipital surface of the skull is eroded and the bone is not preserved. The anterior part of the lateral sinus system, or temporal sinus, is partially reconstructed in blue on figure 3. It extends ventrally and leads to the postglenoid foramen (path for the external jugular vein). Dorsally, it leads to several small foramina indicating vascular communication with the temporal surface. Posteriorly, the occipital sinus runs external to the petrosal bone and opens on the occipital surface at the level of the mastoid foramen (path for the mastoid vein). Ventrally, the petrosal sinus curves slightly forward to the foramen lacerum posterius (internal jugular vein). Just ventral to this flexure, and before the sinus reaches the foramen lacerum posterius, a small posterior sinus corresponding to the condyloid canal opens in the internal aspect of the occipital condyle. Posteroventral to it, on the side of the medulla oblongata, lies the condylar foramen (or hypoglossal foramen) which corresponds to the path of the nerve XII.

Ventromedial to the paraflocullus there is the prominence representing the internal auditory meatus with the cast of the facial nerve (VII), and of the auditory nerve (VIII) located more posteroventrally. The elongated foramen lacerum medium, medial to the internal auditory meatus, connects posteriorly the foramen lacerum posterius by a discrete inferior petrosal sinus. The foramen ovale lies posterolateral to the cavernous sinus and contains the third branch of the trigeminal nerve (V_3_). The surface of the hypophysis or pituitary body cannot be delimited with precision due to deformation and partial preservation of the bone; however, it might be present on the right side of the specimen, medial to the foramen ovale (Figure 3E). The two cavernous sinuses are prominent like in the specimen described by Gazin [35]. They terminate at the position of the posterior aperture to the sphenoidale fissure, and, as suggested by Gazin [35], comprise a complex of vascular structures and cranial nerves (III to VI except V_3_).

### Pattern of *Hyopsodus* cranial endocast compared to that of other archaic ungulates

#### Endocast volume and encephalization quotient

The estimated volume (EV) of the endocast of the specimen of *H. lepidus* (AMNH 143783) described here is 2.79 cm^3^ (sinuses reconstructed in blue on figure 2 not included). Based on external observation of the specimen US 23745 described by Gazin [35], Jerison [17] proposed an endocast volume estimate of 3.2 cm^3^ for *H. miticulus*. The corresponding encephalization quotient (EQ) calculated with an estimated body mass (EM) of 630 g (based on head and body length), is of 0.36 using the equation proposed by Jerison [17] (EQ = EV/0.12(EM)^2/3^), and of 0.49 with the equation of Eisenberg [39] (EQ = EV/0.055(EM)^0.74^). Damuth [40] discussed the problem of estimating body weights of archaic ungulates which greatly differ from extant members of the group. Indeed, the body shape and proportions of *Hyopsodus* are markedly different from those of extant ungulates from which the correlations between dental measurements and body weight are estimated, making it difficult to propose plausible estimates. With its elongated body and relatively short limbs, *Hyopsodus* has been described as a rat-sized animal morphologically close to the weasel [35]. We used different formulas to estimate AMNH 143783 body size: Legendre [41] regressions for ungulates and for mammals, and Damuth [40] regressions for non-selenodont ungulates using m1 area and m1 length. Depending of the formula used, the estimated body weight of AMNH 143783 ranges between 412 and 854 g. Indeed, differences in body weight estimations have important repercussions on EQ estimates (Table 2). Damuth [40] considers that the use of m1 area overestimates the body mass of basal ungulates because they have larger teeth for their body size than do the average modern ones. This assumption gives more credit to the body weight estimate based on the m1 length (412 g). Given the morphological divergence between *Hyopsodus* and extant ungulates, we consider a body weight estimate comprised between 326 g and 412 g, which corresponds to an EQ between 0.48 and 0.41 following the equation of [17], [20], and between 0.59 and 0.70 according to Eisenberg's equation [39]. These EQ values are slightly superior to the estimate of Jerison ([17]; EQ = 0.36) based on a heavier body weight estimate. According to the EQ estimates calculated by Jerison [17] and Radinsky [20], *Hyopsodus* has one of the highest EQ of basal ungulates (Tab. 2): higher values are only observed for the mesonychian *Mesonyx* (“flesh-eater”) and for “advanced ungulates” such as *Homacodon* [20].

**Table 2:** Data for brain size estimate of basal ungulates.

Species	Method/reference	EV (cm^3^)	EBM (Kg)	EQ1	EQ2
*Hyopsodus lepidus* AMNH 143783	Legendre [41] - ungulates	2.79	0.854	0.25	0.34
*Hyopsodus lepidus* AMNH 143783	Legendre [41] - mammalian	2.79	0.326	0.48	0.70
*Hyopsodus lepidus* AMNH 143783	Damuth [40] – m1 area	2.79	0.633	0.31	0.43
*Hyopsodus lepidus* AMNH 143783	Damuth [40] – m1 length	2.79	0.412	0.41	0.59
*Hyopsodus miticulus*	Jerison [17]	3.2	0.630	0.36	0.49
*Actocyon primaevus*	Radinsky [20]	37	29.7–40.8	0.25–0.31	0.33–0.26
*Actocyonides arenae*	Radinsky [20]	12	3.9–5.4	0.32–0.39	0.48–0.38
*Phenacodus primaevus*	Radinsky [20]	35	52.8	0.20	0.20
*Meniscotherium meniscum*	Radinsky [20]	12	6.8–9.1	0.22–0.27	0.32–0.25
*Pleuraspidotherium*	Jerison [17]	6	3.3	0.23	0.26
*Mesonyx obrusidens*	Radinsky [20]	80	35.4–47.2	0.49–0.60	0.62–0.50
*Hyracotherium sp.*	Radinsky [42]	25	9.068	0.46	0.53
*Homacodon vagans*	Radinsky [20]	9.5	0.72–1.01	0.77–0.96	1.33–1.03

EV, endocast volume; EBM, estimated body mass; EQ1, encephalization quotient using Radinsky's [20] equation; EQ2, encephalization quotient using Eisenberg's [39] equation.

#### Development of the neopallium

**Figure 4:**
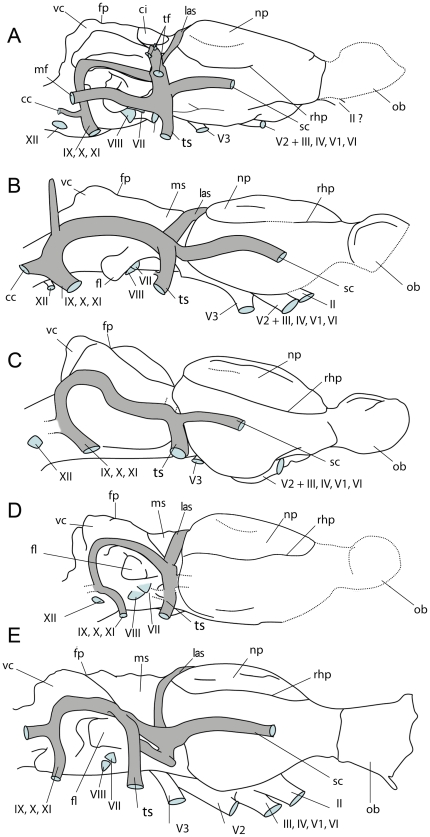
Reconstruction of the lateral view of *Hyopsodus lepidus* compared with other basal ungulates. Reconstructions made after the original cranial endocasts of: (A) *Hyopsodus lepidus* (AMNH 143783), (B) *Pleuraspidotherium* (MNHN CR 252, 963; cast AMNH 39266); (C) *Phenacodus* (AMNH 4369); (D) *Meniscotherium* (AMNH 49082; USNM 19509); (E) *Arctocyon* (MNHN CR 700), modified after Russell and Sigogneau (1965). Not to scale. Abbreviations: same as Figure 2.

By its structure, the neopallium of *Hyopsodus* is rather simple among archaic ungulates. There is no junction between the posterior and anterior rhinal fissures, the location of the latter being even difficult to determine with precision (Figure 3). *Pleuraspidotherium*, *Arctocyon* and *Meniscotherium* have a more clearly marked rhinal fissure (Figure 4), with a junction between the anterior and posterior parts. In *Hyopsodus*, the ventral extension of the neopallium is weak and it does not reach the level of what is interpreted here as a vascular sinus. It is comparable to that of *Pleuraspidotherium* or *Arctocyon*. *Meniscotherium* has a more important development of the neopallium; it even exhibits an anterior inflection of the rhinal fissure that might delineate the frontal lobe ventrally (Figure 4D). This inflection is also observed in early artiodactyls (*Cebochoerus*) and perissodactyls (*Hyracotherium*). The neopallium of *Hyopsodus* is devoid of fissures and has no trace of suprasylvia or a lateral sulcus, it only exhibits a slight inflation of the posterior part of the neopallium. The same morphology is observed in *Meniscotherium* (AMNH 48082; Gazin, 1965), which shows a more important posterior inflation of the neopallium. This is in contrast with the neopallium of *Phenacodus*, *Arctocyon* and *Pleuraspidotherium*, which present clear sulci. *Phenacodus* bears a long lateral sulcus and another small more ventral depression possibly representing the suprasylvia. After verification on the original endocasts (MNHN CR 956, CR 700) *Arctocyon* presents both the lateral and the suprasylvian sulci (accordingly to [31]). *Pleuraspidotherium* only presents one oblique sulcus in a rather posterior and external position (AMNH 39266), interpreted as the suprasylvia by Russell and Sigogneau [31]. In the basalmost perissodactyl *Hyracotherium* (AMNH 55267), there are three well distinct sulci (suprasylvian, ectolateral and lateral, [42]), whereas the simplest artiodactyl endocast presents only two sulci (lateral sulcus and the suprasylvia, [16]). By its proportions, the neopallium of *Hyopsodus* is rather advanced as its antero-posterior extension almost covers half of the total length of the brain.

#### Midbrain exposure

Midbrain exposure has often been correlated with a weak neocortical expansion [43]. It is wide in the pleuraspidotheriid *Pleuraspidotherium*, and the arctocyonids *Artocyon* and *Artocyonides* in which it represents one third of the length of the cerebral hemispheres. In these taxa, however, the corpora quadrigemina do not leave imprints on the skull roof. In the description of the endocast of *Artocyon*, Russel and Sigogneau [31] mentioned that a part of the corpora quadrigemina might be visible on the left side of the endocast (MNHN CR 700); however, there is no trace of this structure on the other specimen (MNHN CRL 956) and its location would be too low and too lateral to correspond to the corpora quadrigemina. *Meniscotherium* also presents a wide exposure of the midbrain. Gazin [34] suggested that the lateral sinuses of *Meniscotherium* might correspond to the corpora quadrigemina, but that their direct continuity with the sagittal sinus rather indicates that it is highly improbable. *Hyopsodus* midbrain exposure is as important as in other archaic ungulates, but its structure is more derived by the presence of inflated inferior colliculi protruding dorsally. To sum up, *Hyopsodus* is the only “basal ungulate” with hypertrophied inferior colliculus known so far.

#### Relative proportions of brain components

**Figure 5:**
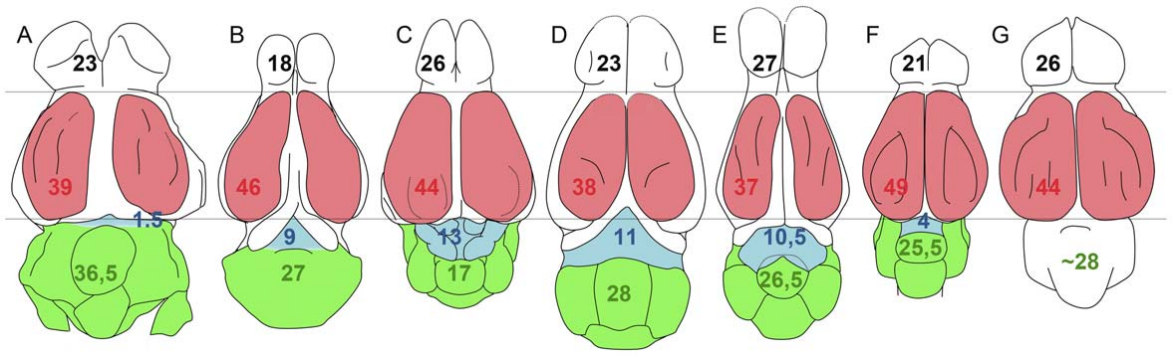
Dorsal view of some basal ungulates. Reconstructions made after the original cranial endocasts of: (A) *Phenacodus* (AMNH 4369), (B), *Meniscotherium* (AMNH 49082), (C) *Hyopsodus* (AMNH 143783), (D) *Pleuraspidotherium* (MNHN CR 252, 963), (E) *Arctocyon* (MNHN CR 700), (F) *Cebochoerus* (MNHN 34–1967), (G) *Hyracotherium* (AMNH 55267). All specimens normalized on the neopallium length. Proportions of the different parts indicated in percent of the total endocast length: black, rhinencephalon; red, neopallium; blue, mesencephalon; green, cerebellum. Not to scale.

Despite their simple lissencephalic structure, the antero-posterior extension of the cerebral hemispheres is important in *Hyopsodus* and represents more than 40% of the total brain length. By the proportions of the different parts of its brain, *Hyopsodus* is closer to “advanced ungulates” like basal perissodactyls (e.g. *Hyracotherium*, [42]) or artiodactyls (e.g. *Cebochoerus*, [16]), than to phenacodontids or arctocyonids (Figure 5). The extension of the neocortex (in red Figure 5) is important compared to that of the cerebellum (in green Figure 4); a feature also observed in “advanced ungulates”. *Hyopsodus* midbrain exposure is as important as in other archaic ungulates, but its structure is more derived. The reduction of the cerebrum relative to the total length of the endocast in *Hyopsodus* is most probably correlated with the development of the inferior colliculi. It is noticeable that the morphology of the vermis itself, with a posterior location of the fissure prima, is primitive [16], despite its reduced antero-posterior extension. *Hyopsodus* presents one of the highest EQs among archaic ungulates, a statement congruent with the derived general proportions of its brain. *Hyopsodus* presents an “advanced version” of the basal ungulate brain pattern, with a mosaic of archaic (large olfactory bulbs, weak ventral expansion of the neopallium, absence of neopallium fissuration) and highly derived (relative reduction of the cerebellum compared to cerebrum; enlargement of the inferior colliculus) characters.

### Paleoecological inference based on cranial endocast morphology

The most significant feature observed in *Hyopsodus* is the clear delineation of the corpora quadrigemina, and especially the large development of the inferior colliculus. The superior colliculus receives major inputs from the retina but is more than just a visual processing structure. One of its major functions is to localize a stimulus and to cause the animal to orient to the stimulus by moving its neck and/or its eyes [44]. In contrast to the role of the superior colliculus within the visual system, the inferior colliculus is the principal source of input to the auditory thalamus, which relays auditory information to the primary auditory cortex [45]. Moreover, the inferior colliculus probably also represents a major output to premotor pathways that initiate or regulate sound evoked motor behavior [46].

While exposure of the midbrain may be a primitive feature of mammals correlated to the weak development of the cerebral hemispheres, it has also been argued that midbrain exposure may result from secondary sensory specialization, especially when the development of the colliculi is involved as in extant and extinct chiropterans (bats, [43]). Such a claim has also been made for the exposure of the colliculi in dermopterans (flying lemurs, [47], [48]). It is noteworthy that the inferior colliculus of *Hyopsodus* is elevated above the midbrain plane which indicates that inferior colliculi are basically exposed because of their development and not because of a lack of posterior extension of the cerebral hemispheres. Gazin [35] argued that the neopallium of *Hyopsodus* was very simple and might not have been able to process complex information. However, in extant mammals, the colliculi have descending projections to the paramedian pontine reticular formation and spinal cord, and thus can be involved in response to stimuli faster than cortical processing would allow. Their development is thus independent from neocortical complexity. In particular, the auditory system is unique among sensory systems with its highly complex network of pathways in the lower brainstem and a significant amount of processing accomplished in the inferior colliculus, prior to the level of the thalamus and the cerebral cortex [49]. The relative size of the inferior colliculus in *Hyopsodus* suggests a high level of development of acoustic reflexes. Development of auditory sense is commonly observed in animals where visual sense is reduced such as in some nocturnal animals or fossorial animals (e.g., [50]).

**Figure 6:**
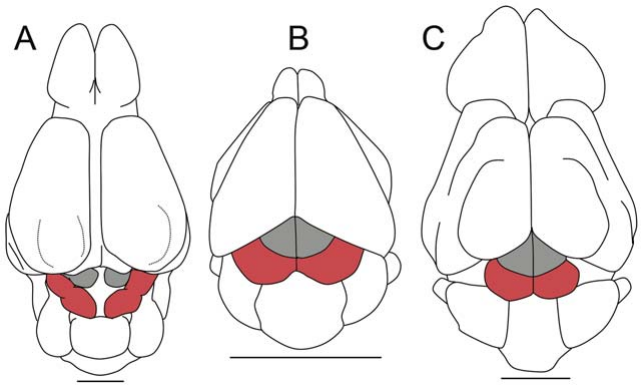
Development of the corpora quadrigemina in (A) *Hyopsodus*, (B) *Rhinolophus hipposideros*, (C) *Tenrec ecaudatus*. Scale bar = 5 mm.

Mammalian echolocation is mostly associated with cetaceans in aquatic environments and bats in aerial environments (e.g., Microchiroptera and Megachiroptera; [51]–[52]). Echolocation, however, has also been demonstrated in terrestrial mammals such as Soricomorpha (shrews) and Tenrecomorpha (tenrecs), mostly in association with subterranean environments or nocturnal habits [53]. The use of low-intensity, short-duration ultrasonic pulses for echolocation has been described in soricid shrews, including the common shrew *Sorex araneus* and the short-tailed shrew *Blarina brevicauda* [54]–[58]. Although discussed, the ecological significance of echolocation in shrews was proposed to aid the search for protective cover or for detecting obstacles in subterranean tunnels. Evidence for echolocation by means of tongue clicks has also been observed in three Tenrecidae both under experimental and natural conditions, and correlated to nocturnal exploration of new environments [53]. Those two insectivorous mammals, although phylogenetically distant (e.g., [59], [60]) present a rather simple brain structure. However, like in bats, both show a well developed inferior colliculus; the latter being largely exposed in the Tenrecidae *Tenrec* (Figure 6) and *Geogale* [61], [62].

Among edentate placentals, the Xenarthra *Chlamyphorus* (pink fairy armadillo, or “*pichiciego*” which means “short-sighted”) has a subterranean life. It shows a huge enlargement of the posterior (inferior) colliculus [43] compared to the optic midbrain colliculi. Edinger [43] parallels the peculiar morphology of the brain of *Chlamyphorus* and its highly derived forelimbs, specialized for digging. Moreover, she relates the large size of the posterior (inferior) colliculus to the unique faculty of *Chlamyphorus* among armadillos to have a “voice” [63], [64]. The fact that this mammal is fossorial suggests that the latter, as shrews and tenrecs, might be another example of underground echolocation in extant terrestrial mammals.

Although no strict relationship between echolocation and the development of inferior colliculus has been demonstrated in extant terrestrial mammals, the inferior colliculus size in *Hyopsodus* could indicate that the latter used terrestrial echolocation to investigate subterranean environment and/or to minimize predation during nocturnal exploration of the environment as concluded for extant shrews [55]–[57].

### Postcranial ecological signal *vs* cranial endocast morphology

*Hyopsodus* was a weasel-sized mammal with a long body, shortened limbs and clawed feet. The postcranium of *Hyopsodus* has been extensively described and figured in Gazin [35] and this animal has either been considered as partly living in trees [65], or adapted to digging or rooting [35] and was likely an herbivorous ground and tree dweller [66]. Gazin interpreted *Hyopsodus* skull shape, dentition and postcranial morphology as clues of rooting and digging habits. Rose [29] describes *Hyopsodus* as having the body shape of a weasel or a prairie dog, and as being capable of climbing or digging but with not apparent specialization for any particular mode of life. Indeed, *Hyopsodus* postcranium is not particularly adapted to digging. The olecranon of the ulna and anterior ungual phalanges are not particularly elongated, the manus is not transformed and the humerus does not show any characteristic specialization related to fossorial ability: the deltopectoral crest is long but not prominent anteriorly, the diaphysis is thin and smooth, and the entepicondyle projects medially more than in *Erinaceus* and *Rhynchocyon*, reaching or overpassing that of *Solenodon*, *Spermophilus*, and Eocene leptictids [67]. However, the scapula bears a very high spine shifted posteriorly, and the elbow stabilization, together with the reduction of supination movements, could indicate some digging abilities for *Hyopsodus*. Rose [67] mentions the following as forelimb traits associated with terrestrial, and possibly digging behaviour: a nearly flat ulnar facet on the radius allowing little supination, separate scaphoid and lunar facets on the distal radius promoting stability at the wrist, and prominent extensor tubercles on the metacarpals and ungual phalanges, all features that can be observed in *Hyopsodus* (see [35] plate 10). As mentioned by Gazin [35], the femur possesses a greater trochanter that does not project proximally beyond the head and a very distal location of the third trochanter, interpreted by this author as an adaptation for abducting the limb, a position consistent with digging. However, in extant mammals, the position of the third trochanter is not necessarily correlated to the extension of the gluteus superficialis it supports, which impedes any functional interpretation of this character. On the other hand, the dorsally prominent tuberosity of the ischion (where the extensors of the hip joint originate) and the well-defined femur trochlea between sharp crests suggest that *Hyopsodus* could move swiftly. The femoral trochlea is better defined in *Hyopsodus* than in *Tenrec*, *Tupaia*, and *Erinaceus*, but less deep anteroposteriorly than in *Rhynchocyon* and Eocene leptictids [67], and the tibia and fibula are not fused. The overall morphology of the postcranial skeleton of *Hyopsodus* suggests a nimble, fast moving animal, with digging abilities and able, then, to live in burrows like small extant mustelids.

Matthews [65] regarded *Hyopsodus* as semi-arboreal. Even if its general morphology and the amplitude of its movements allowed *Hyopsodus* to climb in trees like extant mustelids, its postcranial morphology indicates that it was not particularly adapted to arboreal life: the stabilization of the elbow joint, characterized by the deep trochlea of the humerus, the perforated olecranon fossa, the reduced lateral epicondylar crest and an elongated humeral head and twisted proximal extremity of the humerus, differs greatly from what is observed in extant arboreal mammals such as primates and carnivorans (Argot, pers obs.). The oval shape of the head of the radius, and the reduced supination capabilities suggested by the tight radius-ulna contact, also indicates that *Hyopsodus* was not particularly adapted to arboreal life. Only the medially prominent entepicondyle and shape of the ulna, posteriorly convex, may be found in arboreal mammals.

Morphology and proportions of the endocranial cast of *Hyopsodus* suggest a developed auditory sense, possibly associated with terrestrial echolocation. The overall morphology of the postcranial skeleton of *Hyopsodus*, suggesting a nimble, fast moving animal likely to live in burrows, would be compatible with terrestrial echolocation and/or with nocturnal habits. The occurrence of a highly specialized behavior such as terrestrial echolocation could be put in question by the archaic neocortical structure of *Hyopsodus*. However, the high encephalization quotient of the latter and the relative proportions of the different brain segments, close to more advanced ungulates, make *Hyopsodus* brain outstanding among basal ungulates. Its advanced brain structure might have played a role in the long temporal range of this taxon; *Hyopsodus* persists in the fossil record until the Late Eocene and is among the last “condylarths” to go extinct in North America [38].

## Materials and Methods

This study is based on the complete *Hyopsodus* skull AMNH 143783 housed at the American Museum of Natural History. The specimen consists in a complete cranium only lacking I1-2 on the left side and C, I3-1 on the right side. The mandible preserves a complete dentition. Its posterior part is broken and the angle, as well as the ascending ramus of the mandible, are missing on both sides. The size of the specimen, intermediate between *H. paulus* and *H. minusculus*, and its primitive dental morphology (as described by Flynn [68]; characters include: absence of molarization of the p4, well developed m1–2 hypoconid, absence of strong preprotocrista on P3–4, absence of strong hypocone on M1–2, absence of strong molar lophodonty) allows referring AMNH 143783 to *Hyopsodus lepidus* Matthew, 1909. Both a 3D reconstruction of the dentition (Figure S1) and dental measurements (Table S1) are provided in online supporting information. The exact provenance of this specimen remains uncertain, however, the known temporal range of *Hyopsodus lepidus* is Bridgerian (late Early – early Middle Eocene, [68]). To our knowledge, this skull has never been figured nor described prior to the present study.

The skull AMNH 143783 was scanned with a high resolution CT-scanner at the American Museum of Natural History. The scans resulted in 1300 slices with dimensions of 990 by 1000 pixels. The slices have a 60.83201 µm thickness and are spaced 60.83201 µm apart; two-dimensional slices were reconstructed from X-rays using Vgstudiomax® (version 1.2; VolumeGraphics GmbH, 2004). The 3D segmentation of bone, endocast and sinuses was performed using Aviso® (VSG); endocast volume has been calculated by surface integration. Measurements were taken with Aviso following the protocol of Macrini [69], endocast flexure was measured following the protocol of Macrini et al. [14]. Encephalization quotient was calculated both with the equation defined by Jerison [17] and Radinsky [20]: EQ = EV/0.12 (EM)^0.67^, and with the equation defined by Eisenberg [39]: EQ = EV/0.055 (EM)^0.74^. This latter equation is based on a regression analysis of empirical data from a large sample of extant placental mammal species and confirmed the now widely accepted exponent value of about 0.75 for the scaling of brain size to body size among mammals generally.

## Supporting Information

Figure S1 Digital reconstruction of AMNH 143783 left dentition. A, upper and lower cheek teeth in occlusion, lateral view; B–D, c-m3 in (B) occlusal, (C) lingual, (D) labial views; E–F, C-M3 in (E) occlusal, and (F) lingual views. Scale bar = 5 mm.
(TIF)

Table S1 Dental measurements of AMNH 143783 referred to *Hyopsodus lepidus* (given in mm). Measurements are base don the 3D reconstruction of the dentition.
(DOC)
