# Supplementary figures and images for: Digital Cranial Endocast of Hyopsodus (Mammalia, “Condylarthra”): A Case of Paleogene Terrestrial Echolocation?
Source: PLoS One. 2012 Feb 10;7(2):e30000. doi: 10.1371/journal.pone.0030000 (PMC3277592; doi:10.1371/journal.pone.0030000)

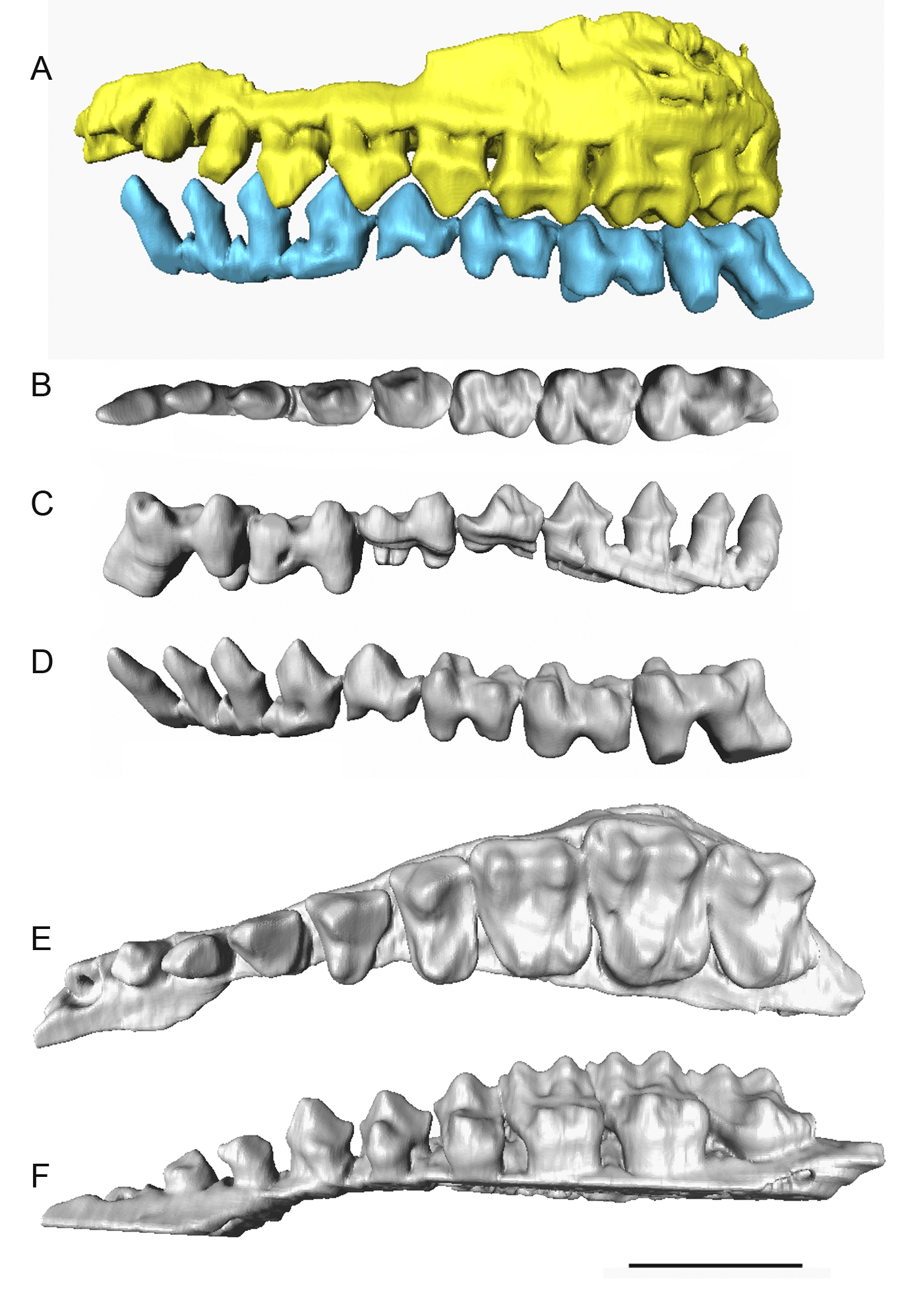

Supplement: Figure S1 — Digital reconstruction of AMNH 143783 left dentition. A, upper and lower cheek teeth in occlusion, lateral view; B–D, c-m3 in (B) occlusal, (C) lingual, (D) labial views; E–F, C-M3 in (E) occlusal, and (F) lingual views. Scale bar = 5 mm. (TIF) [file pone.0030000.s001.tif]
