# Supplementary material for: Digital Cranial Endocast of Hyopsodus (Mammalia, “Condylarthra”): A Case of Paleogene Terrestrial Echolocation?
Source: PLoS One. 2012 Feb 10;7(2):e30000. doi: 10.1371/journal.pone.0030000 (PMC3277592; doi:10.1371/journal.pone.0030000)
Supplement: Table S1 — Dental measurements of AMNH 143783 referred to Hyopsodus lepidus (given in mm). Measurements are base don the 3D reconstruction of the dentition. (DOC) [file pone.0030000.s002.doc]

Table S1 – Dental measurements of AMNH 143783 referred to *Hyopsodus* *lepidus* (given in mm). Measurements are base don the 3D reconstruction of the dentition.

|  | length | width |
| --- | --- | --- |
| C | 1.4 | 1.4 |
| P1 | 1.5 | 1.3 |
| P2 | 2.1 | 1.8 |
| P3 | 2.5 | 2.6 |
| P4 | 2.4 | 3.6 |
| M1 | 3.0 | 3.9 |
| M2 | 3.4 | 4.7 |
| M3 | 3.0 | 4.1 |
| c | 1.4 | 1.2 |
| p1 | 1.4 | 1.1 |
| p2 | 1.8 | 1.3 |
| p3 | 2.4 | 1.5 |
| p4 | 2.8 | 2.0 |
| m1 | 3.1 | 2.5 |
| m2 | 3.3 | 2.8 |
| m3 | 4.1 | 2.7 |
